# Barriers and facilitators to maintaining confidentiality for HIV and AIDS patients in OR Tambo, Eastern Cape

**DOI:** 10.4102/curationis.v48i1.2768

**Published:** 2025-10-31

**Authors:** Ntombesitatu Qotoyi, Agrinette N. Madolo

**Affiliations:** 1 Department of Nursing, Faculty of Health Sciences, Walter Sisulu University, Mthatha, South Africa

**Keywords:** barriers, facilitators, confidentiality, boundaries, HIV and AIDS, primary healthcare, Eastern Cape

## Abstract

**Background:**

Healthcare practitioners are required to keep patient information private, though sharing is sometimes necessary for patient care, risking confidentiality breaches that can compromise privacy rights. Professional nurses lead HIV prevention, treatment, and care services, but issues like loss to follow-up and confidentiality breaches leading patients to move between clinics to hide their status pose challenges.

**Objectives:**

This study explored factors influencing professional nurses’ ability to maintain confidentiality in primary healthcare facilities in OR Tambo, Eastern Cape, aiming to develop intervention strategies.

**Method:**

A qualitative, descriptive, contextual design was used. Nineteen purposively sampled professional nurses participated in semi-structured interviews until data saturation. Interviews were audio-recorded, transcribed verbatim, and thematically analysed using Tesch’s method.

**Results:**

Findings revealed that confidentiality is impacted by resource shortages, limited knowledge, negative attitudes, and inadequate training. Challenges vary across facilities, but nurses recognize the importance of confidentiality. Participants suggested the need for ongoing training on guidelines and consent procedures to uphold confidentiality.

**Conclusion:**

Strengthening confidentiality in HIV care requires comprehensive strategies, including staff empowerment, infrastructure improvements, and initiatives to positively influence staff attitudes.

**Contribution:**

These insights can guide the development of targeted interventions, inform curriculum enhancements, and support future research initiatives.

## Introduction

The Joint United Nations Programme on HIV and AIDS (UNAIDS) ‘95-95-95’ strategy aims for 95% of HIV-infected individuals to be diagnosed by 2025, with 95% of them on ART and 95% achieving sustained virologic suppression. Achieving these targets by 2025 could reduce the HIV epidemic to a low-level endemic disease by 2030 (UNAIDS 2024). It is important to maintain confidentiality for individuals diagnosed with HIV, although it can be compromised by healthcare providers’ task shifting and sharing. Globally, maintaining confidentiality in HIV treatment, care and support involves both successes and challenges, as there are still instances of perceived breaches of confidentiality and limitations to this protection (Kisigo et al. 2020; Nguyen et al. 2024). This empirical study will serve as the foundation for developing a conceptual framework, a training programme and other interventions.

### Problem statement

The study from Limpopo, South Africa, highlights that both patients and nurses view lack of confidentiality as a key barrier to maintaining consistent follow-up among people living with HIV (Modipane, Khoza & Ingersoll 2023). There is a significant concern that patients who are lost to follow-up may later present at hospitals with complications arising from untreated or poorly managed HIV. These observations prompted the design of this study to explore how professional nurses construct boundaries and uphold confidentiality within HIV services, including an examination of the barriers they encounter in maintaining these confidentiality practices.

### Aim of the study

The purpose of the study was to explore the barriers and facilitators of professional nurses to maintain confidentiality for patients with HIV and AIDS.

### Rationale of the study

This study anticipated to enhance the knowledge, skills and attitudes of professional nurses to improve ethical practices in HIV prevention, treatment and care services. It will focus on maintaining patient confidentiality and identifying barriers to confidentiality. Additionally, the study seeks to improve retention in care and reduce HIV transmission, which will be achieved by viral load suppression.

### Conceptual framework

The Information, Motivation, and Behavioural Skills Model (IMB), developed by Fisher, Fisher and Harman (2003), was employed to identify key factors and address barriers related to implementing confidentiality guidelines within HIV prevention, treatment and care. As a behavioural theory, the IMB model emphasises the determinants of human behaviour, suggesting that behaviour is influenced by information, motivation and behavioural skills. According to Meyer and Van Niekerk (2008), behaviourist theories view human behaviour as governed by stimuli, meaning that the information provided by facilitators during in-service training, along with motivational strategies, can enhance the behavioural skills of professional nurses. This, in turn, supports their ability to establish boundaries and uphold confidentiality throughout the HIV treatment cascade. The IMB model was thus adopted to identify data concepts that reveal information gaps, underscoring the need for targeted interventions to equip nurses with the necessary skills to change behaviour and ensure confidentiality is maintained.

## Research methods and design

The research design outlines the plan for the study. The researcher interacts with participants to draw conclusions that meet the research aims and objectives. The research design specifies what will be explored and the methodology used in the study (Brink, van der Walt & van Rensburg 2012). A descriptive and exploratory qualitative research design was employed to examine how professional nurses handle the confidentiality of patient information in HIV management at primary healthcare (PHC) facilities in the OR Tambo district of the Eastern Cape. This study was exploratory because of the limited information available on how professional nurses manage confidentiality in various aspects of HIV management, including counselling and testing, treatment and follow-up care for patients at PHC facilities in OR Tambo.

### Research setting

The study setting involved PHC facilities in OR Tambo district, with community health centres (CHCs) selected because of their service packages and the high number of clients on ART.

### Research population

This study’s general population composed of professional nurses in CHC facilities in OR Tambo district municipality.

### Sampling of PHC facilities

Purposive sampling of the research setting was conducted. The selection of PHC facilities was based on the total number of patients remaining on ART during the study period. Community health centres with the highest volume of patients on ART and the highest number of adults lost to follow-up during a 9-month period (01 January–30 September 2021) were selected. Data were sourced from the District Health Information System (DHIS) and Tier.net reports from the OR Tambo district.

### Sampling method of professional nurses for interviews

Non-probability purposive sampling was used to select 19 professional nurses for interviews (Moule & Goodman 2014). These nurses met specific criteria to provide detailed information about the phenomenon under investigation. The participants had to meet the following criteria to be included in this study.

#### Inclusion criteria

Participants must:

1. Be professional nurses employed by the Department of Health in OR Tambo district, Eastern Cape.
2. Have at least 1 year of experience in HIV prevention, treatment and care programme.
3. Have a good understanding of English to participate in the interviews.

#### Exclusion criteria

1. Nurses on contract and partners not employed by the Department of Health in OR Tambo were excluded.
2. Nurses with less than 1 year of experience in HIV care were excluded.

### Sample size

This study used a qualitative approach with a sample of 19 professional nurses, following the saturation principle until no new information emerged (Patten & Newhart 2018).

### Data collection

Data collection involves systematically gathering information relevant to research, with interviews being a possible method (Polit & Beck 2012). In-depth, individual, semi-structured one-on-one interviews were conducted with an interview guide to gather data from participants. The pre-determined open-ended questions guided the researcher to ensure consistency across interviews (Grove et al. 2015). The semi-structured interviews were recorded, transcribed verbatim after obtaining consent to record from participants (Moule & Goodman 2014). With no affiliation to the OR Tambo district, the researcher employed bracketing to reduce bias. After obtaining permission to conduct the study in the OR Tambo district, the researcher contacted sub-district managers to establish relationships and discuss participant involvement in the interviews. This included informing management about the planned data collection dates.

### Data analysis

Data analysis included reducing, organising and interpreting the collected data (Burns & Grove 2016). Tesch’s eight steps of thematic data analysis as described in Creswell (2009) were used as a structured approach for analysing textual data. Here is a summarised overview:

1. The researchers transcribed and organised interview data into categories based on sources to understand the full context.
2. They repeatedly read the data, noting meanings and adding comments to capture insights.
3. Topics were listed, clustered and arranged into main and subcategories.
4. Topics were abbreviated into codes placed alongside relevant text segments to facilitate identification of new categories and codes.
5. Categories were labelled with descriptive phrases for clarity.
6. Abbreviations and codes were standardised and organised alphabetically.
7. A preliminary analysis involved collecting data for each category in one place and documenting it.
8. Finally, they interpreted the data by grouping categories and subcategories, developing a tentative structure of nurses’ experiences.

Following the analysis of the data by the researcher, themes and sub-themes were developed. The verbatim transcripts/raw data were then sent to an experienced independent coder in qualitative research for co-analysis. The themes and sub-themes identified by the researcher closely matched those identified by the independent coder. These themes and sub-themes were subsequently discussed, and a consensus was reached on the final themes to be used in the study.

### Measures to ensure trustworthiness

Polit and Beck (2017) recommend implementing measures to ensure trustworthiness throughout the research process to maintain the validity and reliability of the research. Trustworthiness is characterised by evaluating the truth value of qualitative research. Botma et al. (2010) identified four key aspects to ensure trustworthiness in qualitative research: credibility, transferability, dependability and confirmability. Trustworthiness indicates how reliable and truthful the findings are perceived to be. Credibility reflects truth value, transferability relates to applicability, dependability pertains to consistency and confirmability addresses neutrality (Brink et al. 2012).

### Ethical considerations

Research ethics stem from professional codes of conduct and national laws (De Vos et al. 2011). This research followed principles of beneficence, respect for human dignity and justice, ensuring protection of human rights like self-determination, privacy, anonymity, confidentiality and fair treatment.

Permission for the study was obtained from the Research Ethics Committee of Walter Sisulu University (reference number: 021/2022), the Easten Cape Department of Health and OR Tambo health district management. Participants were informed about the study’s purpose and objectives before consenting. Consent forms were used, and participants were told they could withdraw at any time without penalty (Brink et al. 2012).

## Research findings

Out of 19 professional nurses interviewed, 16 were females and 3 were males, with HIV management experience ranging from 3 to 31 years. The findings are summarised into 3 themes and 9 sub-themes from data analysis.

### Theme 1: Participants detailed the barriers and facilitators to maintain confidentiality for patients with HIV

The interviewed participants revealed that confidentiality is affected by a lack of resources and knowledge regarding confidentiality. The participants further detailed the facilitators of confidentiality, including the use of guidelines to facilitate adherence and the significance of consent as a facilitator to construct boundaries for maintaining confidentiality. The literature revealed that PHC services in South Africa offer essential first-contact healthcare, but nurses in PHC clinics face many challenges in their practice environments. These challenges include nursing shortages, lack of resources, inadequate support, lack of leadership and higher levels of job dissatisfaction (Rabie, Coetzee & Klopper 2016). The participants mentioned that staff shortages are a serious concern, as professional nurses offer guidance in their leadership roles in HIV services. The following quote confirms this:

‘I think sometimes eehh … [*sigh*] the staff shortage when there is no professional nurse to guide.’ (P13, female, 49 years old)

Professional nurses hold a leadership role in nursing care and are accountable for service delivery omissions. Leadership is essential to guide health professionals and develop them to meet global needs (ed. Clarke 2014). This manifested in the following sub-themes that emerged from this overarching theme.

#### Sub-theme 1.1: Lack of resources

The participants identified the lack of resources as the primary barrier. Specifically, they highlighted issues such as insufficient staff, inadequate space, the absence of personnel to cover for sick staff and a shortage of consulting rooms. These resource constraints were viewed as significant challenges to maintaining confidentiality in HIV services. The space deficiencies often result in patient information being overheard by others. To clarify the issue of staff shortage, the following participants explained how it affects confidentiality:

‘I think sometimes eehh … the staff shortage when there is no professional nurse to guide. At times we have a staff nurse, and certain things are not done. This affect confidentiality because sometimes there are things a patient wants to tell a professional nurse and a junior nurse does not know and may end up discussing it in front of everyone. Maybe someone who has more experience and knowledge may know how to maintain confidentiality. The one with less knowledge may ask someone else and not know how to approach.’ (P13, female, 49 years old)

The issue of staff shortage was extended to the challenge of records management, which contributes to breaches of confidentiality indirectly, as shared by participants in the following extracts:

‘Concerning records and files most of the patients are not followed properly when using different files for maternity and other conditions as sometimes are misfiled due to staff shortage.’ (P16, female, 35 years old)

‘The first one is that in the facility we have shared access to the files and some of the workers are not professionals. This shortage of staff is a problem in that case.’ (P19, female, 58 years old)

The reasons contributing to the shortage of staff at health facilities are as follows: a shortage of qualified healthcare workers has been found as one of the significant issues facing developing countries and is likely to be a critical impediment to achieving healthcare targets. The shortage of health workers is exacerbated by the emigration of skilled healthcare professionals to other countries. Absenteeism is common among healthcare workers (Koto & Maharaj 2016).

The shortage of consulting rooms and shared spaces compromised privacy, causing discomfort to both nurses and patients. Nurses often had to ask patients or colleagues to leave the room temporarily during consultations. Mobile screens were used as an alternative to create privacy; however, some participants noted challenges because of the lack of available screens:

‘… Even the consulting rooms are short as nurses are sharing consulting rooms, if there is a problem with the client the professional nurse ask another client to step aside then professional nurse will talk to that client. If there is something more than then treatment.’ (P6, female, 50 years old)

Similarly, another participant elaborated on how they improvise to ensure privacy when no screens are available:

‘Yha … [*sigh*] we do work like in the consulting room we are two professional nurses working in that room. When there is a need for confidentiality, we let the other nurse to go out and the other nurse to finish with that patient. We talk in a soft voice not to be loud so that they can’t listen to what you are saying. We do have mobile screens so that we at least cover so that they cannot see each other as patients.’ (P19, female, 58 years old)

Participants often compromised privacy and confidentiality to keep queues moving. If privacy was maintained, clients might have to wait longer, or nurses would need to leave, delaying services. The findings echo those of other studies, which found that inadequate infrastructure results in long queues and increases the workload of health workers (Koto & Maharaj 2016). Participants’ practices differed depending on the availability of space, as illustrated by the following:

‘One challenge that we have is the space, we usually share the consulting room. You find that there is an area where there are four people sharing the space and use screens. Someone may hear what is said to the other one.’ (P8, male, 35 years old)

‘Some of the barriers. we have some few consulting rooms. There is little or no space to communicate with the patient alone. On Wednesdays both teams are in the facility. Sometimes you find out that there are two professional nurses and Enrolled Nurses. Though they are professional nurses the client do not feel comfortable.’ (P13, female, 49 years old)

‘The challenge is the space infrastructure is not conducive to maintain confidentiality, but we try by all means. There are no screens, and we work as two professional nurses. When we want to push the queue when it is full we call maybe two clients at a time. Its where we gonna see that we have this problem and the other is even negative. Then there are barriers there. We try to excuse the other one who is negative to treat this one. Imenza [it makes the patient] suspicious, the one who is excluded.’ (P4, female, 43 years old)

The sub-theme highlighted that participants acknowledged infrastructural issues as contributing factors to unintentional confidentiality breaches by healthcare personnel. Overall, resource limitations such as insufficient staff, inadequate space and a lack of consulting rooms serve as significant barriers that compromise confidentiality within healthcare settings.

#### Sub-theme 1.2: Lack of knowledge regarding confidentiality

Participants described addressing their knowledge gaps through on-the-job training and informal learning, emphasising the need for structured training programmes to enhance understanding of confidentiality principles. They highlighted that ongoing education could improve their confidence and competence in maintaining patient privacy, especially for non-professional staff who may lack formal training. Such initiatives are crucial to ensure that all healthcare workers, regardless of their role or experience level, are equipped with the necessary knowledge to uphold confidentiality standards effectively. Ceylan and Çetinkaya (2020) reaffirm that nurses’ understanding of patient confidentiality directly influences how they implement confidentiality practices in their care. This underscores the importance of comprehensive training and education to ensure nurses are well-informed about confidentiality principles, ultimately promoting better patient trust and privacy. Participants explained how they addressed knowledge gaps as follows:

‘We try by all means to minimise the information or the critical part of the information because we just give them what is on their level of scope of practice. If they are CHWs [*community health workers*] they have to go and fetch the client. We just tell them to fetch the client without divulging the status of the client because sometimes they must found out about the status. The lay counsellors should not tell the status of the client to other people. There are confidentiality trainings they attend, those training bind them to keep confidentiality.’ (P9, female, 47 years old)

‘We use the staff that is non-professional. Those people are being monitored, and we limit the information that we give to maintain confidentiality when they go for community services like track and tracing.’ (P8, male, 35 years old)

‘We do not divulge the information except to the nurses and on tier.net, in that data capturers room whereby they are storing files there is nobody allowed because anybody can go there and search for information. Information can be found and professionalism not maintained and may capture information if there is a fight and send and escalate that this one so and so I saw that you are on treatment.’ (P2, female, 35 years old)

‘… Data capturer kuba uzakufaka information yakhe, after capturer izakuvulela I file after that they capture the information ekule file bazaku coder bafaka ku Tier net. [*Data capturer will open a file and capture the information. Then will code the client and capture in tier.net*] Data capturers are trained in confidentiality by the health professionals.’ (P5, female, 60 years old)

Healthcare professionals in this study expressed concerns about the confidentiality knowledge of CHWs. These concerns align with findings from a study conducted in Swaziland, where community participants reported mistrust of cadres, citing inadequate training on confidentiality issues, particularly for services related to stigmatised conditions (Geldsetzer et al. 2017).

#### Sub-theme 1.3: Guidelines for confidentiality to facilitate adherence

The participants described employing guidelines aimed at building trust and encouraging patients to remain in care, underscoring the importance of confidentiality and trust in HIV service delivery. The South African National Policy on HIV Counselling and Testing emphasises the importance of maintaining full confidentiality of HIV test information, only disclosing details with the individual’s informed consent (van Dyk 2013). Despite this, stigma remains a significant barrier, influencing individuals’ decisions to seek testing, stay in care and adhere to treatment. The following participants shared their experiences:

‘ …[*W*]e emphasise that we need to write down. We are using the guidelines and the policies.’ (P3, female, 49 years old)

‘We are being more cautious about results. What we want to gain from our patients is trust so that they will be able to communicate freely with the nurse without fear, knowing that the information discussed in the consulting room end there. There is no leakage so that patients get trust to nurses.’ (P16, female, 35 years old)

Confidentiality is ensured during follow-ups on missed appointments. A participant detailed the following process:

‘The form has the ticks we just tell the CHWs [*community health workers*] to go and fetch the person. If she ask what is it for, you just say tell her that she is called to the clinic to be seen by a nurse. So they do come some stay from the far locations. We send the outreach teams using the cars for the department but even when they do not have stickers they have the red number plates. When we visit the patients we follow the protocol. We first go to the chief home to ask for permission to access a certain household. We leave the car in the chief’s home and the chief will take you to the household. I think confidentiality is not breached because in that household you do not go with the department car.’ (P9, female, 47 years old)

The guidelines and protocols provided essential guidance for professional nurses to uphold confidentiality throughout all stages of the HIV care cascade, from treatment initiation to follow-up. By adhering to these standards, nurses could better protect patient privacy, foster trust and enhance patient engagement and retention in care.

#### Sub-theme 1.4: The significance of consent as a facilitator of confidentiality

The participants demonstrated a solid understanding of informed consent, emphasising its importance in ethical healthcare practice. The review also highlighted the different types of consent discussed by professional nurses, such as explicit, implied and verbal consent, underscoring their relevance in various clinical scenarios. Informed consent allows patients to make decisions regarding testing, treatment, home visits and support. The study conducted in Ethiopia among HIV and AIDS patients on ART concluded that healthcare workers should actively encourage patients to make informed decisions about the type of care and information they wish to share. This approach helps to reduce breaches of confidentiality by empowering patients and respecting their autonomy (Bayisa et al. 2022). The participants detailed their procedures for securing various forms of informed consent in the following statements:

‘We maintain confidentiality by letting the patient make decisions and sign informed consent. Then those who are negative we offer prevention strategies.’ (P8, male, 35 years old)

‘Verbal consent for treatment and the written consent is for testing. If the patient does not accept initiation of treatment the refusal is signed to be a proof that it was offered. The one who accept initiation of treatment we offer treatment and talk about shared confidentiality that there will be other team members that may come concerning the treatment that the information will not be only between the two of us that is why it is called shared confidentiality.’ (P15, male, 43 years old)

The participants further detailed the instances where they obtain verbal consent to grant the patients an opportunity to choose. The process is explained as follows:

‘We ensure that the patient does consent. We initiate ARVs. We ask the patient if they consent to home visits. We ask if the patient prefer to take the treatment in the facility or elsewhere. Then the patient goes to the pharmacy with the prescription.’ (P13, female, 49 years old)

‘First ask the patient does she accept the status. At home who is the support system because what is important is the support then initiate treatment. Even before you initiate treatment you need to get consent from the patient and understand what is going on. You do not make decisions for patients.’ (P16, female, 35 years old)

The above statements indicate that there are standard practices to obtain consent from testing, treatment and support services. To promote the choice, consent is obtained when stable clients are due for decanting to adherence clubs. Informed consent has been used as confidentiality agreement between the healthcare providers and clients in HIV care. Participants further explained the process of obtaining consent for adherence clubs, which is illustrated as follows:

‘First thing we have HIV testing and counselling whereby once the patient is tested and found to be positive we have shared confidentiality whereby we obtain verbal consent from the client to share information with other health providers in the facility. As for treatment we have adherence clubs before the patient goes to the clubs we obtain consent from the patient so that we share the information with those clubs. They just give limited information as possible.’ (P7, female, 31 years old)

The sub-theme explained that the nurses understand the need for informed consent; however, because of a high number of support programme that may affect confidentiality, continuous trainings may benefit the nurses to implement the guidelines.

### Theme 2: Participants related the challenges regarding breaches of confidentiality

Theme two (2) presents the challenges in maintaining confidentiality and the practices that lead to breaches. Professional nurses are expected to help others deal with moral challenges but may also experience similar moral challenges regarding breaches of confidentiality because of lack of professional guidance (Ligtenberg, Molewijk & Stolper 2024). The ethical dilemmas surrounding HIV confidentiality reflect the complex intersection of medical, legal and social challenges (Rossouw 2024). Participants in the study experienced challenges regarding breaches of confidentiality, as revealed in the following sub-themes: breach of confidentiality related to non-professional staff’s inexperience and approaches to dealing with breaches of confidentiality.

#### Sub-theme 2.1: Breach of confidentiality related to non-professional staff’s inexperience

Participants openly and earnestly discussed the use of non-professional staff for follow-up and retention in HIV care as a significant challenge. The World Health Organization (WHO) advocates for the utilisation of CHWs as non-professional personnel in HIV management. When provided with appropriate education, competencies, supervision and support, CHWs can safely and effectively perform a broad range of roles – including preventive, diagnostic, treatment and care services – and facilitate linkages with the wider health system, where permitted by national policy (WHO 2021). However, barriers such as inadequate support, resource shortages, insufficient training and poor monitoring have been identified as obstacles to effective community-based HIV care (Ngcobo et al. 2022). To address these issues, recommendations include tackling HIV-related stigma, updating CHW training curricula and strengthening supervision mechanisms (Ngcobo et al. 2022). The following quotes from participants highlight different categories of these non-professional workers and their roles:

‘… By doing counselling and testing using lay counsellors and professional nurses. Before the patient start treatment, the professional nurse who is going to start a treatment start with counselling. The Community Health Workers involved once the client is not taking treatment well. Once the Viral load is suppressed after 3 months the client is enrolled to CCMDD [*Central Chronic Medicine Dispensing and Distribution*] and adherence on treatment is emphasised to the client.’ (P6, female, 50 years)

Participants highlighted the issue of inexperience as follows:

‘One of the lay counsellors disclosed the status of the mother to the child and ended up not taking treatment and said to the mother you must take the treatment as you are the one who gave me HIV. The lay counsellor was called, and the issue was sorted.’ (P12, female, 51 years old)

The inexperience of non-professional health workers is exacerbated by non-compliance with treatment guidelines and careless behaviour, as attested in the following quote:

‘The supporting partner’s staff. I took it as breach of confidentiality as that headman got to know what these parcels are for. They did not attend the adherence club. They arrive in the school or headman to drop. They did not wait for the clients to come and fetch their treatment.’ (P10, male, 38 years old)

The behaviour described above aligns with findings from a study conducted in hospitals supporting the University of Cordoba, which highlighted that healthcare worker behaviours contribute to breaches of confidentiality. Most breaches are unintentional, stemming from ignorance about behaviours that jeopardise patient confidentiality (Beltran-Aroca et al. 2016).

Participants further detailed instances of spontaneous gossip among CHWs, which indicate inexperience. This is evidenced by the following quote:

‘Let’s say you are offering counselling to a person and she [*community health worker*] may come and tell you that this person may test positive because “I know that there is someone who is positive in that family.” We tell her that she must never share that information because you are trying to convince the person to test. You might be declaring something that is not allowed to be known by someone else.’ (P8, male, 35 years old)

Professional nurses guide and mentor non-professional healthcare workers on boundary construction and confidentiality issues. However, the issue was highlighted as a challenge because of the unavailability of clear guidelines. To extend the concerns, another participant adds:

‘They are staying [*community health workers*] in the same community these patients are coming from. There is that fear to breaking their confidentiality.’ (P19, female, 58 years old)

The study conducted in Swaziland highlighted a key concern regarding CHWs’ potential breach of confidentiality. Participants expressed that because CHWs are members of the same community as their clients, there is a risk that they might share sensitive health information with others within the community who know the client. This familiarity could undermine trust in the confidentiality of health disclosures, which is crucial for effective healthcare delivery. The findings, as reported by Geldsetzer et al. (2017), underscore the importance of addressing community members’ perceptions and ensuring proper training and protocols are in place to maintain confidentiality in community-based health services. Another challenge is the access of records by non-vetted cadres from the supporting partners, which can lead to a conflict of interest as detailed by the following participant:

‘There is this girl who had a boyfriend who was newly appointed as data capturer by the NGO [*non-governmental organisation*] she did not know that he was working in this facility, he saw that she is taking treatment when he gave her the file. She was concerned about her status, she was scared on how he will react at home. I felt that maybe if we have a way of us as nurses to capturer but we are unable to do. We try by all means to explain to them that they must not say that they saw that someone is HIV positive.’ (P7, female, 31 years old)

Healthcare facilities need a proper system to ensure that vetted cadres handle records to avoid personal interest and conflict. Healthcare workers need orientation on how to construct boundaries to separate private life to professional duties in the workplace.

#### Sub-theme 2.2: Approaches to dealing with a breach of confidentiality

The participants shared different approaches used by management in healthcare facilities to address breaches of confidentiality. These breaches are often reported as complaints, as explained by the following participant:

‘Yes, personally I had one complaint. I was looking for a patient that was no longer getting medication and then when I asked why she has stopped taking medication, she said that the community health worker that is working in her community was sharing information on why she was looking for a specific person and also when she delivered medication she showed others that I am taking this medication so that people can see. To manage it I reported to the facility manager then we called the person involved and emphasized importance of confidentiality and the impact it might have on the patient side. We called the patient and promised to strengthen our confidentiality, and the patient continued to take medication well.’ (P8, male, 35 years old)

‘They are usually called by the disciplinary committee within the facility.’ (P15, male, 43 years old)

‘We implement consequence management if you breach the confidentiality. There was a case of ill treatment, and the patient did not honour appointment date for treatment. The sister NGO took the treatment and not found the patient. They left the treatment with one of the family members. The patient did not disclose yet to the family members. The patient came to face the facility for not honouring confidentiality clause. We had the meeting to redress the patient the one who took the medicine had to apologise and had to sign some forms … a warning.’ (P19, female, 58 years old)

The participants described methods used to handle breaches of confidentiality. These methods align with the South African Nursing Council (SANC) prescripts for managing unprofessional conduct (Kaseke 2023). Disciplinary action must be taken against professionals who fail to do so. According to Odia (2014), professional bodies may also take disciplinary action against erring physicians, as disclosing confidential information constitutes infamous professional conduct.

### Theme 3: Suggestions to improve confidentiality in HIV

The participants provided suggestions for improving confidentiality in HIV management. Training, workshops, infrastructure improvement and focusing on staff attitudes were highly recommended. Inadequate training refers to the gap between actual training and the skills, abilities, competencies, attitudes and knowledge needed to perform specific functions (ed. Clarke 2014). Training aims to enhance knowledge, skills and behaviour to achieve objectives or goals, improving competency and care quality (ed. Clarke 2014).

#### Sub-theme 3.1: Training and workshops are prerequisites to improve confidentiality

Participants suggested in-service training and workshops for all staff categories. Continuous education was emphasised, with a focus on raising awareness about potential breaches of confidentiality and the importance of protecting patients’ privacy:

‘Refresher training, for example, I was trained in 2015 and there are new developments. In-service training and workshops.’ (P10, male, 38 years old)

‘We can improve by attending in-service trainings conducted by health and refresher trainings as we seem to forget. I did not attend any training. I only attended ART [*antiretroviral therapy*] guidelines that were talking about medication. There was nothing on confidentiality. Maybe if there is an in-service training to help us to find a strategy to help the patient on how to disclose to family members. It is difficult for them. Maybe she is from Cape Town and default the treatment whilst the relatives do not know that she is on treatment. If we can be trained on how the patient can tell the family members.’ (P14, female, 54 years old)

In-service training refers to training provided within the healthcare service to improve professional knowledge, skills, values and attitudes according to healthcare demands (Muller & Bester 2016). The participant suggested extending training to all cadres of healthcare workers, as indicated in the following quote:

‘The suggestion is that we keep on doing in-service trainings for health professionals, the community health workers, and the lay counsellors. We have data capturers who work in collection of files. We have got the general assistants. To improve confidentiality, we have to align with the training of HIV management links like the knowledge hub; foundation for professional development; South African HIV clinician society and the knowledge training units. It would assist in promotion of confidentiality for health professionals if done as the course.’ (P19, female, 58 years old)

A study conducted among healthcare workers to assess their knowledge and perceptions regarding ethics, confidentiality and medico-legal issues revealed high knowledge levels and positive perceptions. However, regular training was recommended to update knowledge and ensure continuous improvement in healthcare delivery quality (Barnie et al. 2015).

The participants expressed support for confidentiality training. This aligns with findings from a study conducted in Indonesia, which indicated that confidentiality is not adequately taught and is often breached in practice (Nixon et al. 2014).

#### Sub-theme 3.2: Improvement of infrastructure and sufficient resources to deliver optimal healthcare for patients with HIV

Participants indicated that infrastructure needs attention to maintain confidentiality. The need for additional consulting rooms was raised during the interviews. Insufficient resources hinder the delivery of optimal healthcare. The following participants attested to this:

‘The care for HIV is poor because of infrastructure. Government can give us consulting room as others bayayiva [*hear*] everything as we are using screens.’ (P12, female, 51 years old)

‘More working space can improve confidentiality to ensure that the patient has one-on-one to share information with the nurse. I see in other facilities that they have man’s corner and youth corner.’ (P13, female, 49 years old)

‘At least if we can have another container as the building is not big enough to add consulting rooms.’ (P4, female, 43 years old)

The situation in Limpopo, South Africa, highlights significant infrastructural challenges impacting HIV service delivery. Nurses reported a shortage of consulting rooms, which has led clinics to utilise post-natal wards for HIV counselling sessions (Tshililo et al. 2019). This inadequate space compromises client confidentiality and may hinder effective counselling. Crowley and Stellenburg (2014) emphasise that essential HIV care, treatment services and infrastructural support are vital for delivering quality healthcare, including providing sufficient consulting rooms to ensure privacy. Similarly, findings from a Tanzanian study reinforce these concerns, identifying infrastructural limitations as key obstacles to high-quality HIV services. The research underscores that adequate infrastructure is critical not only for maintaining client confidentiality but also for reducing the workload on healthcare providers, thereby improving overall service quality (Iseselo et al. 2024). Significant infrastructure challenges hinder confidentiality in HIV/AIDS services. Moving between consultation rooms for privacy is time-consuming and may alert others. Healthcare providers should take care to ensure confidentiality.

#### Sub-theme 3.3: Focus on staff attitudes and behaviours to improve confidentiality and patient care

Interview participants emphasised that negative staff attitudes towards patients affect both team performance and the maintenance of confidentiality. These attitudes not only impact clients but also impact fellow healthcare providers, as reflected in poor report-handling practices and limited participation in HIV-related in-service training.

The participants attest:

‘Sometimes you know the nurses if you give report they say its for this one and others [*nurses*] do not come. They think if its chronic it is not for them and will send those who do HIV. The attitude is poor because even if you stop the person when she speak about the patient information she will continue even during lunch.’ (P12, female, 51 years old)

‘We need workshops especially on attitudes have to understand and respect our patients. We need to get information on new regulations and in-service training on confidentiality.’ (P16, female, 35 years old)

‘Yes, the patient complained, and I think it was not addressed properly. The nurse talked rudely to a patient who missed the date. The patient came to the office but someone who was in the office that day did not call the nurse. She addressed the patient alone and did not call the nurse to come and apologise to the client so that she can come again. The manager said this need to be addressed so that, that nurse may stop causing problems of attitude to the patients.’ (P2, female, 35 years old)

‘There are barriers like when you have to write down not to talk about patient information. Some of us are talkative that is why we emphasise that we need to write down.’ (P3, female, 49 years old)

To echo this, studies show that while health professionals in resource-limited settings generally have good knowledge about confidentiality, their attitudes towards maintaining it are limited (Tegegne et al. 2022). Additionally, research in Zimbabwe found that poor confidentiality and negative attitudes from healthcare providers are key barriers to HIV service utilisation (Tafuma et al. 2018). The professional nurse’s suggestions will assist in the development of strategies to minimise the barriers and improve confidentiality in HIV care.

### Recommendations

To enhance the management of confidentiality and disclosure issues, it is recommended to capacitate professional nurses with problem-solving skills specifically related to breaches of confidentiality and treatment support. Ongoing in-service training and workshops should be implemented regularly, as suggested by study findings and participant feedback, to keep nurses updated on best practices and ethical considerations. Additionally, addressing resource shortages is crucial; primary healthcare management should prioritise resource allocation to improve service delivery. Furthermore, conducting studies on healthcare professionals’ understanding of boundary construction within collaborative teams can contribute valuable insights to the existing body of knowledge, promoting better teamwork and ethical practices in healthcare settings.

## Conclusion

The participants highlighted various procedures and challenges associated with maintaining confidentiality in HIV care services. Although practices are not standardised – partly because of differing facility demographics – participants acknowledged the importance of confidentiality. They rely on existing guidelines and policies as key facilitators to uphold confidentiality standards. However, concerns were raised regarding the involvement of CHWs and lay counsellors, who are often inexperienced and lack sufficient knowledge about boundary construction and confidentiality issues. To ensure proper information sharing among health professionals, professional nurses obtain explicit client consent before sharing any health information. These findings underscore the need for standardised protocols, ongoing training and capacity-building to address confidentiality challenges effectively.
